# Supplementary material for: Smooth Interpolating Curves with Local Control and Monotone Alternating Curvature
Source: Comput Graph Forum. 2022 Oct 6;41(5):25–38. doi: 10.1111/cgf.14600 (PMC9827861; doi:10.1111/cgf.14600)
Supplement: Supplementary file 1 — Supplement Material [file CGF-41-25-s001.zip › Local-Smooth-Interpolating-MonoCurvature/extern/clothoids/docs/api-matlab/class_a00084.html]

Class ClothoidList — Clothoids v2.0.9

### Navigation

- index
- toc
- next
- previous
- Clothoids »
- MATLAB API »
- Class ClothoidList

# Class ClothoidList¶

- Defined in File ClothoidList.m

## Inheritance Relationships¶

### Base Type¶

- `public CurveBase` (Class CurveBase)

## Class Documentation¶

class ClothoidList : public CurveBase¶
:   Public Functions

    function ClothoidList()¶
    :   Create a new C++ class instance for the clothoid list object

        **Usage:**

        ```
        ref = ClothoidCurve()
        ```

        **On output:**

        - `ref`: reference handle to the object instance

    function is\_type( ignoredArg)¶

    function reserve( self, N)¶
    :   Reserve memory for `N` segments

        **Usage:**

        ```
        ref.reserve( N );
        ```

    function save( self, fname)¶
    :   Save the clothoid list to a file

    function load( self, varargin)¶
    :   Load the clothoid list from a file (check consistency of the readed segments)

        **Usage:**

        ```
        ref.load( filename );
        ref.load( filename, tol );
        ```

        `filename` : file name to be read `tol` : tolerance used to check consistency

    function push\_back( self, varargin)¶
    :   Append a curve to the clothoid list

        **Usage:**

        ```
        ref.push_back( obj ); % mode 1
        ref.push_back( kappa0, dkappa, L ); % mode 2
        ref.push_back( x0, y0, theta0, kappa0, dkappa, L ); % mode 3
        ```

        **Mode 1**

        - `obj` : curve to be appended can be one of

          - *LineSegment*
          - *BiArc*
          - *BiarcList*
          - *CircleArc*
          - *ClothoidCurve*
          - *ClothoidList*
          - *PolyLine*

        **Mode 2**

        - `kappa0` : initial curvature of the appended clothoid
        - `dkappa` : derivative of the curvature of the appended clothoid
        - `L` : length of the the appended clothoid

        **Mode 3**

        - `x0`, `y0` : initial position of the appended clothoid, the builded clothoid will be translated to the and of the actual clothoid list
        - `theta0` : initial curvature of the appended clothoid
        - `kappa0` : initial curvature of the appended clothoid
        - `dkappa` : derivative of the curvature of the appended clothoid
        - `L` : length of the the appended clothoid

    function push\_back\_G1( self, varargin)¶
    :   Append a curve to the clothoid list

        **Usage:**

        ```
        ref.push_back( x1,y1,theta1 ); % mode 1
        ref.push_back( x0,y0,theta0,x1,y1,theta1); % mode 2
        ```

        **Mode 1**

        Build a clothoid arc using final point and angle of the clothoid list and

        - `x1`, `y1` : final point
        - `theta1` : final angle

        the builded clothoid is appended to the list

        **Mode 2**

        Build a clothoid arc using the data

        - `x0`, `y0` : initial point
        - `theta0` : initial angle
        - `x1`, `y1` : final point
        - `theta1` : final angle

        the builded clothoid is appended to the list

    function get( self, k)¶
    :   Get the clothoid at position `k`. The biarc is returned as a clothoid object or the data defining the clothoid.

        ```
        [ x0, y0, theta0, kappa0, dkappa, L ] = ref.get(k);
        C = ref.get(k);
        ```

    function append( self, lst)¶
    :   Append a curve to a clothoid list.

        ```
        C = LineSegment( ... );
        ...
        C = Biarc( ... );
        ...
        C = CircleArc( ... );
        % ....
        ref.append(C); % append biarc
        ```

    function getSTK( self)¶
    :   Return curvilinear coordinates, angle and curvature at node point for the clothoid list

        ```
        [ s, theta, kappa ] = ref.getSTK();
        ```

        - `s` curvilinear coordinates nodes
        - `theta` angles at nodes
        - `kappa` curvature at nodes

    function getXY( self)¶
    :   Return xy-coordinates at node points for the clothoid list

        ```
        [ x, y ] = ref.getXY();
        ```

        - `x` x-coordinates at nodes
        - `y` y-coordinates at nodes

    function numSegments( self)¶

    function build\_3arcG2( self, x0, y0, theta0, kappa0, )¶

    function build\_3arcG2fixed( self, s0, x0, y0, theta0, kappa0, )¶

    function build\_2arcG2( self, x0, y0, theta0, kappa0, )¶

    function build\_CLC( self, x0, y0, theta0, kappa0, )¶

    function build\_G1( self, x, y, varargin)¶
    :   Given a list of xy-coordinates at node points build a clothoid list. If third argument (`angle`) is not present angles are estimated internally.

        ```
        ref.build_G1( x, y );
        ref.build_G1( x, y, theta );
        ```

        - `x` x-coordinates at nodes
        - `y` y-coordinates at nodes
        - `theta` angle at nodes

    function build( self, x0, y0, theta0, s, kappa)¶
    :   Given a list of curvilinear coordinated and curvatures at nodes build a G2 clothoid list. Initial position and angle must be set to determine a unique clothoid list.

        ```
        ref.build( x0, y0, theta0, s, kappa );
        ```

        - `x0` initial x-coordinates
        - `y0` initial y-coordinates at nodes
        - `theta0` initial angle
        - `s` list of curvilinear coordinates
        - `kappa` list of curvatures at nodes

    function build\_theta( self, x, y)¶
    :   Given a list of xy-coordinates at node points build a guess of angles for the clothoid list.

        ```
        theta = ref.build_theta( x, y );
        ```

        - `x` x-coordinates at nodes
        - `y` y-coordinates at nodes

    function build\_raw( self, x, y, abscissa, theta, kappa)¶

    function make\_closed( self)¶

    function make\_open( self)¶

    function is\_closed( self)¶

    function deltaTheta( self)¶

    function deltaKappa( self)¶

    function closestPointBySample( self, qx, qy, ds)¶

    function distanceBySample( self, qx, qy, ds)¶

    function closestSegment( self, qx, qy)¶

    function closestPointInRange( self, qx, qy, ibegin, iend, varargin)¶

    function closestPointInSRange( self, qx, qy, s\_begin, s\_end, varargin)¶
    :   Find closest point of a Clothoid list given a s-range

        ```
        [ icurve, x, y, s, t, iflag, dst ] = ...
           ref.closestPointInSRange( qx, qy, s_begin, s_end, varargin );

        [ icurve, x, y, s, t, iflag, dst ] = ...
           ref.closestPointInSRange( qx, qy, s_begin, s_end, varargin, 'ISO' );

        [ icurve, x, y, s, t, iflag, dst ] = ...
           ref.closestPointInSRange( qx, qy, s_begin, s_end, varargin, 'SAE' );

        res = ref.closestPointInSRange( qx, qy, s_begin, s_end, varargin );
        res = ref.closestPointInSRange( qx, qy, s_begin, s_end, varargin, 'ISO' );
        res = ref.closestPointInSRange( qx, qy, s_begin, s_end, varargin, 'SAE' );

        %
        % res is a struct with field
        %
        % res.icurve = number of the segment with the projected point
        % res.x      = projected x
        % res.y      = projected y
        % res.s      = curvilinear coordinate of the projection
        % res.t      = normal curvilinear coordinate ('ISO' or 'SAE' convenction)
        % res.iflag  = 1 OK -1 projection failed
        % res.dst    = point curve distance
        %
        ```

    function s\_to\_index( self, s)¶

    function export\_table( self, filename)¶

    function export\_ruby( self, filename)¶

    function info( self)¶

    function find\_coord1( self, x, y, varargin)¶

    function plot( self, varargin)¶
    :   Plot the clothoid list

        **Usage:**

        ```
        ref.plot();
        ref.plot( npts );

        fmt1 = {'Color','blue','Linewidth',2}; % first arc of the biarc
        fmt2 = {'Color','red','Linewidth',2};  % second arc of the biarc
        ref.plot( npts, fmt1, fmt2 );
        ```

        - `npts`: number of sampling points for plotting
        - `fmt1`: format of the odd clothoids
        - `fmt2`: format of the even clothoids

    function plot\_offs( self, offs, npts, varargin)¶
    :   Plot the clothoid list with offset

        **Usage:**

        ```
        ref.plot_offs( offs, npts );

        fmt1 = {'Color','blue','Linewidth',2}; % first arc of the biarc
        fmt2 = {'Color','red','Linewidth',2};  % second arc of the biarc
        ref.plot_offs( offs, npts, fmt1, fmt2 );
        ```

        - `npts`: number of sampling points for plotting
        - `fmt1`: format of the first arc
        - `fmt2`: format of the second arc
        - `offs`: offset used in the plotting

    function plotCurvature( self, npts, varargin)¶
    :   Plot the curvature of the clothoid list

        **Usage:**

        ```
        ref.plotCurvature( npts );

        fmt1 = {'Color','blue','Linewidth',2};
        fmt2 = {'Color','red','Linewidth',2};
        ref.plotCurvature( npts, fmt1, fmt2 );
        ```

        - `npts`: number of sampling points for plotting
        - `fmt1`: format of the first arc
        - `fmt2`: format of the second arc

    function plotAngle( self, npts, varargin)¶
    :   Plot the angle of the clothoid list

        **Usage:**

        ```
        ref.plotAngle( npts );

        fmt1 = {'Color','blue','Linewidth',2};
        fmt2 = {'Color','red','Linewidth',2};
        ref.plotAngle( npts, fmt1, fmt2 );
        ```

        - `npts`: number of sampling points for plotting
        - `fmt1`: format of the first arc
        - `fmt2`: format of the second arc

    function plotNormal( self, step, len)¶
    :   Plot the normal of the clothoid list

        **Usage:**

        ```
        ref.plotNormal( step, len );
        ```

        - `step`: number of sampling normals
        - `len`: length of the plotted normal

    function saveSampled( self, filename, ds)¶
    :   Save the clothoid list sampled on a file

        **Usage:**

        ```
        ref.saveSampled( filename, ds );
        ```

        - `filename`: file name
        - `ds`: sample point every `ds`

        the file is of the form

        ```
        X Y THETA
        0 0 1.2
        ...
        ...
        ```

    function saveClothoids( self, filename)¶
    :   Save the clothoid list on a file as a list of segments

        **Usage:**

        ```
        ref.saveClothoids( filename, ds );
        ```

        - `filename`: file name
        - `ds`: sample point every `ds`

        the file is of the form

        ```
        x0 y0 theta0 kappa0 dk  L
        0  0  1.2    0.0    0.1 2
        ...
        ...
        ```

    function obj\_handle( self)¶
    :   Return the `pointer` of the interbal stored c++ object

        **Usage**

        ```
        obj = ref.obj_handle();
        ```

    function copy( self, C)¶
    :   Make of copy of a curve object

        **Usage**

        ```
        ref.copy( C );
        ```

        where `C` id the curve object to be copied.

    function bbox( self, varargin)¶
    :   Return the bounding box of the curve object

        **Usage**

        ```
        [ xmin, ymin, xmax, ymax ] = ref.bbox( C );
        [ xmin, ymin, xmax, ymax ] = ref.bbox( C, offs );
        [ xmin, ymin, xmax, ymax ] = ref.bbox( C, offs, 'ISO' );
        [ xmin, ymin, xmax, ymax ] = ref.bbox( C, offs, 'SAE' );
        ```

        - xmin: x minimum coordinate of the bounding box
        - ymin: y minimum coordinate of the bounding box
        - xmax: x maximum coordinate of the bounding box
        - ymax: y maximum coordinate of the bounding box

        **Optional Arguments**

        - offs: offset of the curve used in the bbox computation
        - ’ISO’/’SAE’: use ISO or SAE orientation of the normal for offset computation

    function translate( self, tx, ty)¶
    :   Translate the curve by `(tx,ty)`

        **Usage**

        ```
        ref.translate( tx, ty );
        ```

    function trim( self, smin, smax)¶
    :   Cut the curve at the curvilinear parameter `smin` up to `smax`

        **Usage**

        ref.trim( smin, smax );

    function rotate( self, angle, cx, cy)¶
    :   Rotate the curve by angle `angle` around point `(cx, cy)`

        **Usage**

        ```
        ref.rotate( angle, cx, cy );
        ```

    function reverse( self)¶
    :   Reverse the direction of travel of the curve.

        **Usage**

        ```
        ref.reverse();
        ```

    function scale( self, sc)¶
    :   Scale the curve by factor `sc`

        **Usage**

        ```
        ref.scale( sc );
        ```

    function changeOrigin( self, newX0, newY0)¶
    :   Translate the curve in such a way the origin is at `(newX0,newY0`.

        **Usage**

        ```
        ref.changeOrigin( newX0, newY0 );
        ```

    function evaluate( self, s, varargin)¶
    :   Evaluate the curve at curvilinear coordinate `s`. Argument `s` may be a vector for multiple evaluations.

        **Usage**

        ```
        [ x, y, theta, kappa ] = ref.evaluate( s );
        [ x, y, theta, kappa ] = ref.evaluate( s, offs );
        [ x, y, theta, kappa ] = ref.evaluate( s, offs, 'ISO' );
        [ x, y, theta, kappa ] = ref.evaluate( s, offs, 'SAE' );
        ```

        **Optional Arguments**

        - offs: offset of the curve used in computation
        - ’ISO’/’SAE’: use ISO or SAE orientation of the normal for offset computation

    function eval( self, varargin)¶
    :   Evaluate the curve at curvilinear coordinate `s`. Argument `s` may be a vector for multiple evaluations.

        **Usage**

        ```
        XY = ref.eval( s );
        XY = ref.eval( s, offs );
        XY = ref.eval( s, offs, 'ISO' );
        XY = ref.eval( s, offs, 'SAE' );

        [X,Y] = ref.eval( s );
        [X,Y] = ref.eval( s, offs );
        [X,Y] = ref.eval( s, offs, 'ISO' );
        [X,Y] = ref.eval( s, offs, 'SAE' );
        ```

        **Optional Arguments**

        - offs: offset of the curve used compiutation
        - ’ISO’/’SAE’: use ISO or SAE orientation of the normal for offset computation

        **Output**

        - XY: matrix `2 x n` of the evaluated points
        - X: vector of the x-coordinates of the evaluated points
        - Y: vector of the y-coordinates of the evaluated points

    function eval\_D( self, varargin)¶
    :   Evaluate the first derivatives of the curve at curvilinear coordinate `s`. Argument `s` may be a vector for multiple evaluations.

        **Usage**

        ```
        XY = ref.eval_D( s );
        XY = ref.eval_D( s, offs );
        XY = ref.eval_D( s, offs, 'ISO' );
        XY = ref.eval_D( s, offs, 'SAE' );

        [X,Y] = ref.eval_D( s );
        [X,Y] = ref.eval_D( s, offs );
        [X,Y] = ref.eval_D( s, offs, 'ISO' );
        [X,Y] = ref.eval_D( s, offs, 'SAE' );
        ```

        **Optional Arguments**

        - `offs`: offset of the curve used in computation
        - ’ISO’/’SAE’: use ISO or SAE orientation of the normal for offset computation

        **Output**

        - `XY`: matrix `2 x n` of the evaluated points
        - `X`: vector of the x-coordinates of the evaluated point derivatives
        - `Y`: vector of the y-coordinates of the evaluated point derivatives

    function eval\_DD( self, varargin)¶
    :   Evaluate the second derivatives of the curve at curvilinear coordinate `s`. Argument `s` may be a vector for multiple evaluations.

        **Usage**

        ```
        XY = ref.eval_DD( s );
        XY = ref.eval_DD( s, offs );
        XY = ref.eval_DD( s, offs, 'ISO' );
        XY = ref.eval_DD( s, offs, 'SAE' );

        [X,Y] = ref.eval_DD( s );
        [X,Y] = ref.eval_DD( s, offs );
        [X,Y] = ref.eval_DD( s, offs, 'ISO' );
        [X,Y] = ref.eval_DD( s, offs, 'SAE' );
        ```

        **Optional Arguments**

        - `offs`: offset of the curve used in computation
        - ’ISO’/’SAE’: use ISO or SAE orientation of the normal for offset computation

        **Output**

        - `XY`: matrix `2 x n` of the evaluated points
        - `X`: vector of the x-coordinates of the evaluated point derivatives
        - `Y`: vector of the y-coordinates of the evaluated point derivatives

    function eval\_DDD( self, varargin)¶
    :   Evaluate the third derivatives of the curve at curvilinear coordinate `s`. Argument `s` may be a vector for multiple evaluations.

        **Usage**

        ```
        XY = ref.eval_DDD( s );
        XY = ref.eval_DDD( s, offs );
        XY = ref.eval_DDD( s, offs, 'ISO' );
        XY = ref.eval_DDD( s, offs, 'SAE' );

        [X,Y] = ref.eval_DDD( s );
        [X,Y] = ref.eval_DDD( s, offs );
        [X,Y] = ref.eval_DDD( s, offs, 'ISO' );
        [X,Y] = ref.eval_DDD( s, offs, 'SAE' );
        ```

        **Optional Arguments**

        - `offs`: offset of the curve used in computation
        - ’ISO’/’SAE’: use ISO or SAE orientation of the normal for offset computation

        **Output**

        - `XY`: matrix `2 x n` of the evaluated points
        - `X`: vector of the x-coordinates of the evaluated point derivatives
        - `Y`: vector of the y-coordinates of the evaluated point derivatives

    function theta( self, s)¶
    :   Evaluate the angle of the curve at curvilinear coordinate `s`. Argument `s` may be a vector for multiple evaluations.

        **Usage**

        ```
        theta = ref.theta( s );
        ```

    function theta\_D( self, s)¶
    :   Evaluate the angle derivatives (curvature) of the curve at curvilinear coordinate `s`. Argument `s` may be a vector for multiple evaluations.

        **Usage**

        ```
        theta = ref.theta_D( s );
        ```

    function theta\_DD( self, s)¶
    :   Evaluate the angle second derivatuve of the curve at curvilinear coordinate `s`. Argument `s` may be a vector for multiple evaluations.

        **Usage**

        ```
        theta = ref.theta_DD( s );
        ```

    function theta\_DDD( self, s)¶
    :   Evaluate the angle third derivative of the curve at curvilinear coordinate `s`. Argument `s` may be a vector for multiple evaluations.

        **Usage**

        ```
        theta = ref.theta_DDD( s );
        ```

    function kappa( self, s)¶
    :   Evaluate the curvature of the curve at curvilinear coordinate `s`. Argument `s` may be a vector for multiple evaluations.

        **Usage**

        ```
        theta = ref.kappa( s );
        ```

    function kappa\_D( self, s)¶
    :   Evaluate the curvature derivative of the curve at curvilinear coordinate `s`. Argument `s` may be a vector for multiple evaluations.

        **Usage**

        ```
        theta = ref.kappa_D( s );
        ```

    function kappa\_DD( self, s)¶
    :   Evaluate the curvature second derivative of the curve at curvilinear coordinate `s`. Argument `s` may be a vector for multiple evaluations.

        **Usage**

        ```
        theta = ref.kappa_DD( s );
        ```

    function xyBegin( self)¶
    :   Evaluate initial point of the curve.

        **Usage**

        ```
        [ x0, y0 ] = ref.xyBegin();
        ```

    function xyEnd( self)¶
    :   Evaluate final point of the curve.

        **Usage**

        ```
        [ x1, y1 ] = ref.xyEnd();
        ```

    function xBegin( self)¶
    :   Evaluate initial x-coordinate of the curve.

        **Usage**

        ```
        x0 = ref.xBegin();
        ```

    function xEnd( self)¶
    :   Evaluate final x-coordinate of the curve.

        **Usage**

        ```
        x1 = ref.xEnd();
        ```

    function yBegin( self)¶
    :   Evaluate initial y-coordinate of the curve.

        **Usage**

        ```
        y0 = ref.yBegin();
        ```

    function yEnd( self)¶
    :   Evaluate final y-coordinate of the curve.

        **Usage**

        ```
        y1 = ref.yEnd();
        ```

    function thetaBegin( self)¶
    :   Evaluate initial angle of the curve.

        **Usage**

        ```
        theta = ref.thetaBegin();
        ```

    function thetaEnd( self)¶
    :   Evaluate final angle of the curve.

        **Usage**

        ```
        theta = ref.thetaEnd();
        ```

    function kappaBegin( self)¶
    :   Evaluate initial curvature of the curve.

        **Usage**

        ```
        kappa0 = ref.kappaBegin();
        ```

    function kappaEnd( self)¶
    :   Evaluate final curvature of the curve.

        **Usage**

        ```
        kappa1 = ref.kappaEnd();
        ```

    function length( self, varargin)¶
    :   Return the length of the curve.

        **Usage**

        ```
        length = ref.length();
        ```

    function points( self)¶

    function bbTriangles( self, varargin)¶
    :   Evaluate the bounding box triangles of curve.

        **Usage**

        ```
        [P1,P2,P3] = ref.bbTriangles();
        [P1,P2,P3] = ref.bbTriangles(max_angle,max_size);
        [P1,P2,P3] = ref.bbTriangles(max_angle,max_size,offs);
        [P1,P2,P3] = ref.bbTriangles(max_angle,max_size,offs,'ISO');
        [P1,P2,P3] = ref.bbTriangles(max_angle,max_size,offs,'SAE');
        ```

        **Optional Arguments**

        - `max_angle`: maximum curve angle variation admitted in a triangle
        - `max_size`: maximum triangles size
        - `offs`: offset of the curve used in computation
        - ’ISO’/’SAE’: use ISO or SAE orientation of the normal for the offset

        **Output**

        - `P1`: `2 x n` matrix with the first points of the triangles
        - `P2`: `2 x n` matrix with the second points of the triangles
        - `P3`: `2 x n` matrix with the third points of the triangles

    function closestPoint( self, qx, qy, varargin)¶
    :   Evaluate the point at minimum distance of another point on the curve. `qx` and `qy` may be vectors so that the return values are vectors too.

        **Usage**

        ```
        [ x, y, s, t, iflag, dst ] = ref.closestPoint( qx, qy );
        [ x, y, s, t, iflag, dst ] = ref.closestPoint( qx, qy, offs );
        [ x, y, s, t, iflag, dst ] = ref.closestPoint( qx, qy, offs, 'ISO' );
        [ x, y, s, t, iflag, dst ] = ref.closestPoint( qx, qy, offs, 'SAE' );
        ```

        **Optional Arguments**

        - offs: offset of the curve used in computation
        - ’ISO’/’SAE’: use ISO or SAE orientation of the normal for the offset

        **Output**

        - `x`, `y`: Point at minimum distance from `(qx,qy)` on the curve.
        - `s`, `t`: Curvilinear coordinates of the point `(qx,qy)`.
        - `iflag`: `iflag < 0` some error in computation, iflag >0 is the numer of segment containing the point at minimum distance.
        - `dst`: point curve distance.

    function distance( self, qx, qy, varargin)¶
    :   Evaluate the distance of a point `(qx,qy)` to the curve. `qx` and `qy` may be vectors so that the return values are vectors too.

        **Usage**

        ```
        dst = ref.distance( qx, qy );
        dst = ref.distance( qx, qy, offs );
        dst = ref.distance( qx, qy, offs, 'ISO' );
        dst = ref.distance( qx, qy, offs, 'SAE' );
        ```

        **Optional Arguments**

        - `offs`: offset of the curve used in computation
        - ’ISO’/’SAE’: use ISO or SAE orientation of the normal for the offset

    function collision( self, OBJ, varargin)¶
    :   Check if two curve collide.

        **Usage**

        ```
        ok = ref.collision( obj );
        ok = ref.collision( obj, offs, offs1 );
        ok = ref.collision( obj, offs, offs1, 'ISO' );
        ok = ref.collision( obj, offs, offs1, 'SAE' );
        ```

        **Optional Arguments**

        - `offs`, `offs1`: offset of the curves used in computation
        - ’ISO’/’SAE’: use ISO or SAE orientation of the normal for the offsets

    function intersect( self, OBJ, varargin)¶
    :   Intersect two curves.

        **Usage**

        ```
        [s1,s2] = ref.intersect( obj );
        [s1,s2] = ref.intersect( obj, offs, offs1 );
        [s1,s2] = ref.intersect( obj, offs, offs1, 'ISO' );
        [s1,s2] = ref.intersect( obj, offs, offs1, 'SAE' );
        ```

        - `s1`: curvilinear coordinates of the intersections on the first curve
        - `s2`: curvilinear coordinates of the intersections on the second curve

        **Optional Argument**

        - `offs`, `offs1`: offset of the curves used in computation
        - ’ISO’/’SAE’: use ISO or SAE orientation of the normal for the offsets

    function find\_coord( self, x, y)¶
    :   Get the curvilinear coordinates of the point `(x,y)`

        **Usage:**

        ```
        [s,t] = ref.find_coord( x, y );
        ```

        - `s`: curvilinear coordinate along the curve
        - `t`: curvilinear coordinate along the normal of the curve

    function yesAABBtree( self)¶
    :   Activate the use of AABB three in intersection/collision computations

    function noAABBtree( self)¶
    :   Deactivate the use of AABB three in intersection/collision computations

    function plotTBox( self, P1, P2, P3, varargin)¶
    :   Plot a triangle BBOX

        **Usage:**

        ```
        ref.plotTBox( P1, P2, P3 );
        ref.plotTBox( P1, P2, P3, 'Color', 'red' );
        ```

    function plotBBox( self, varargin)¶
    :   Plot the bounding box of the curve

        **Usage:**

        ```
        ref.plotBBox();
        ref.plotBBox('Color', 'red' );
        ```

    function plotTriangles( self, varargin)¶
    :   Plot the covering triangles of the curve

        **Usage:**

        ```
        ref.plotTriangles()
        ref.plotTriangles('red','FaceAlpha', 0.5);
        ```

### Quick search

### Table of Contents

- Matlab Interface Manual
- C++ API
- MATLAB API

«
hide menu

menu
sidebar
»

### Navigation

- index
- toc
- next
- previous
- Clothoids »
- MATLAB API »
- Class ClothoidList

© Copyright 2021, Enrico Bertolazzi and Marco Frego.
Created using Sphinx 4.2.0.
